# AKK-derived outer membrane vesicles alleviate indomethacin-induced mucin secretion reduction in LS174T cells by inhibiting endoplasmic reticulum stress

**DOI:** 10.3389/fmolb.2024.1418876

**Published:** 2024-11-07

**Authors:** Lijun Zhang, Shuang Ma, Huixi Liang, Xin Chen, Jingwen Zhao

**Affiliations:** ^1^ Department of Gastroenterology and Hepatology, Tianjin Medical University General Hospital, Tianjin, China; ^2^ Tianjin Key Laboratory of Digestive Diseases, Tianjin Institute of Digestive Diseases, Tianjin, China

**Keywords:** AKK-derived outer membrane vesicles, indomethacin, goblet cells, MUC2, endoplasmic reticulum stress

## Abstract

**Introduction:**

AKK-derived outer membrane vesicles (AKK-OMVs) have shown potential in modulating intestinal mucosal immunity by increasing the number of intestinal goblet cells. However, it remains unclear whether AKK-OMVs can directly regulate MUC2 secretion in goblet cells exposed to indomethacin *in vitro* and the underlying mechanisms involved.

**Methods:**

The abnormal mucin secretion model in LS174T cells was established using indomethacin, with treatment including *Akkermansia muciniphila* (AKK) supernatant, AKK-OMVs, and extracellular vesicle removal supernatant. The effects of these treatment on MUC2 expression were observed. Transcriptomic sequencing analysis was used to explore the underlying regulatory mechanisms, which were further validated through qRT-PCR and western blotting.

**Results:**

The treatment with AKK supernatant and AKK-OMVs alleviated the indomethacin-induced reduction in MUC2 secretion in goblet cells. Mechanistically, transcriptomic analysis showed that the gene expression associated with endoplasmic reticulum (ER) stress were upregulated after indomethacin treatment in LS174T cells. This suggests that AKK-OMVs, as the active component in the supernatant, improved MUC2 expression by inhibiting ER stress.

**Conclusion:**

AKK-OMVs can directly stimulate goblet cells to promote MUC2 secretion, providing potential for further *in vivo* studies to confirm their protective effects against indomethacin-induced intestinal injury.

## 1 Introduction

Extracellular vesicles (EVs) are lipid bilayer-enclosed structures that carry a variety of biomolecules, including DNA, RNA, proteins, and lipids, and are secreted by virtually all cell type (Toyofuku et al., 2023). These vesicles play crucial roles in intercellular communication by transporting their cargo between cells. In bacteria, two main types of EVs are recognized: membrane vesicles (MVs) from Gram-positive bacteria and outer membrane vesicles (OMVs) from Gram-negative bacteria (Domínguez Rubio et al., 2022). OMVs have attracted considerable attention due to their potential biomedical applications, such as in vaccines, drug delivery, and immunomodulation (Li et al., 2020). They are naturally produced during bacterial growth and are involved in various physiological processes, including the modulation of host immune responses and the maintenance of microbial homeostasis (Hu et al., 2024).

*Akkermansia muciniphila* (AKK), a Gram-negative bacterium, is particularly noted for its beneficial role in gut health (Cani et al., 2022). It is a prominent mucin-degrading bacterium that resides in the mucus layer of the intestinal epithelium, where it contributes to maintaining intestinal homeostasis and strengthening the intestinal barrier (Wang et al., 2023). The OMVs produced by AKK are known to regulate intestinal mucosal responses and influence the composition of the intestinal microbiota, thereby promoting the integrity of the intestinal barrier (Chelakkot et al., 2018). Research has shown that these OMVs can enhance mucus production by goblet cells, stimulate immune responses, and mitigate inflammation, making them potential candidates for therapeutic applications aimed at improving gut health (Wang et al., 2023; Zhao et al., 2024).

Nonsteroidal anti-inflammatory drugs (NSAIDs), such as indomethacin, are widely used for their anti-inflammatory and analgesic effects. However, NSAIDs are also associated with significant gastrointestinal side effects, including damage to the intestinal epithelial cells and disruption of the mucus barrier (Zhu et al., 2023). NSAIDs can interact with cell membranes, altering their biophysical properties and compromising their integrity. This leads to increased permeability and damage to the intestinal mucosa, which ultimately results in inflammatory damage and disruption of the protective mucus layer (Anthony, 1993). Indomethacin, in particular, has been shown to cause mucosal injury in both animal models and humans, impairing the intestinal barrier and reducing the secretion of MUC2, a critical mucin protein secreted by goblet cells (Bai et al., 2023).

Goblet cells play a pivotal role in the secretion of mucins, particularly MUC2, which forms the backbone of the intestinal mucus layer (Gustafsson and Johansson, 2022). This layer acts as a physical barrier that protects the intestinal epithelium from pathogens and mechanical stress. *In vitro* models, such as LS174T cells, have been extensively used to study goblet cell function and the regulation of mucin secretion, given that these cells secrete large amounts of MUC2 (Pelaseyed et al., 2014). In this study, we established an indomethacin-induced model of abnormal MUC2 secretion using LS174T cells to investigate the protective effect of AKK-derived OMVs on MUC2 secretion. Furthermore, we explored the underlying mechanisms by which AKK-OMVs may modulate endoplasmic reticulum (ER) stress and restore MUC2 secretion in the context of NSAID-induced injury. Our investigation contributes to the growing body of evidence supporting the beneficial role of microbial-derived OMVs in maintaining gut homeostasis and suggests that AKK-OMVs could serve as a potential therapeutic intervention to mitigate NSAID-induced intestinal damage.

## 2 Material and methods

### 2.1 Preparation of supernatant, and AKK-OMVs

The *A. muciniphila* strain (CICC 24917) was obtained from the China Center of Industrial Culture Collection (CICC) and stored as a liquid culture in cryovials at −80°C. The strain was inoculated in Brain Heart Infusion Broth (HB8297-1, Hopebio) and constant anaerobic conditions at 37°C for 48 h. The bacterial culture was centrifuged at 10,000 × *g* for 60 min at 4°C to collect the supernatant, which was then filtered through a 0.22 µm filter to remove impurities. The AKK supernatant was ultracentrifuged at 1,50,000 rpm for 2 h at 4°C. After centrifugation, the supernatant was discarded, and the pellet was resuspended in phosphate-buffered saline (PBS). This was followed by a second ultracentrifugation under the same conditions for another 2 h. After discarding the supernatant again, the pellet containing OMVs was resuspended in PBS to obtain the OMVs stock solution, which was then filtered through a 0.22 µm filter and stored at −80°C. AKK-OMVs were then characterized by Nanoparticle Tracking Analysis (NTA) and Transmission Electron Microscopy (TEM) for size and morphology analysis.

### 2.2 Cell model

The LS174T cell line was cultured in MEM complete medium supplemented with 10% fetal bovine serum, 1% penicillin-streptomycin, and 1% non-essential amino acids in an incubator at 37°C with 5% CO₂, with a passage ratio of 1:2 to 1:3 every 2 days.

The cells were divided into the following experimental groups: blank control (NC), indomethacin group (INDO, with 500 μM indomethacin for 12 h), Indomethacin + 1:20 AKK supernatant group (INDO + sAKK1, with 1:20 AKK supernatant treatment for 24 h and indomethacin for 12 h), the indomethacin + 1 μg/mL AKK-OMVs group (INDO + aAKK1, with 1 μg/mL AKK-OMVs for 12 h) and the indomethacin + 1:50 AKK supernatant group (INDO + sAKK2, with 1:50 AKK supernatant for 24 h and indomethacin for 12 h), as well as the indomethacin + 2 μg/mL AKK-OMVs group (INDO + aAKK2, with 2 μg/mL AKK-OMVs for 12 h).

### 2.3 Real-time quantitative polymerase chain reaction (qRT-PCR)

According to the manufacturer’s instructions, Trizol (Vazyme) was used to extract RNA from mouse small intestine tissue and LS174T cells. Reverse transcription into cDNA was performed using HiScript III RT SuperMix for qPCR (+ gDNA wiper) (Vazyme). Real-time quantitative PCR analysis was carried out with ChamQ Universal SYBR qPCR Master Mix (Vazyme). The expression levels of target gene mRNAs were calculated using the ΔΔCT method. The primer sequences used in this study were synthesized by GENEWIZ Biotechnology Company, with the specific sequences for each gene listed in Table 1.

**TABLE 1:** Primer sequence.

Gene		Human
MUC2	forward	ACTCTCCACACCCAGCATCATC
	reverse	GTGTCTCCGTATGTGCCGTTGT
CHOP	forward	GGTATGAGGACCTGCAAGAGGT
	reverse	CTTGTGACCTCTGCTGGTTCTG
GRP78	forward	CTGTCCAGGCTGGTGTGCTCT
	reverse	CTTGGTAGGCACCACTGTGTTC
XBP1	forward	AAGTTCTGCTTCTGTCGGGG
	reverse	GGCTGGTAAGGAACTGGGTC
GAPDH	forward	GTCTCCTCGACTTCAACAGCG
	reverse	ACCACCCTGTTGCTGTAGCCAA

### 2.4 Western blot

Samples were lysed in a buffer containing RIPA lysis buffer, protease inhibitor (Solarbio, P1260), and phosphatase inhibitor (Solarbio, P0100), then placed on ice for 30 min. Afterward, they were centrifuged at 4°C at 13,000 × *g* for 10 min to remove cell debris. Protein concentrations were determined using a BCA kit (Solarbio, PC0020). Equal amounts of protein from each lysate were separated by SDS-PAGE and subsequently analyzed by western blotting. Blots were blocked using a protein-free rapid blocking solution for 10 min, then incubated with primary antibodies (1:1,000) at 4°C overnight in TBST. After washing three times with TBST (9 min each), the blots were incubated with HRP-conjugated secondary antibodies (1:5,000) in TBST at room temperature for 1 h. Protein expression was visualized using the Image Lab detection system. The primary antibodies used in this study were as follows: CHOP (Cell Signaling, 2895), GRP78 (ABclonal, A23453), XBP1s (ABclonal, A17007), and MUC2 (Abcam, ab272692).

### 2.5 Transcriptome sequencing analysis

Total RNA from the samples was isolated and purified using TRIzol (Thermo Fisher, 15596018) according to the manufacturer’s instructions. The quantity and purity of the total RNA were assessed using a NanoDrop ND-1000 spectrophotometer (Wilmington, DE, United States), and RNA integrity was evaluated with a Bioanalyzer 2100 (Agilent, CA, United States). mRNA was specifically captured using oligo (dT) magnetic beads (Dynabeads Oligo (dT), cat. 25-61005, Thermo Fisher, United States) targeting the PolyA (polyadenylate) tail. The captured mRNA was fragmented at high temperature using the NEBNextR Magnesium RNA Fragmentation Module (cat. E6150S, NEB, United States). CDNA synthesis from the fragmented RNA was carried out using Invitrogen SuperScriptTM II Reverse Transcriptase (cat. 1896649, Thermo Fisher, CA, United States). The double-stranded library was treated with UDG enzyme (NEB, cat. M0280, MA, United States) and amplified via PCR, resulting in a chain-specific library with a fragment size of 300 ± 50 bp. Finally, paired-end sequencing (PE150 mode) was performed using the Illumina NovaSeqTM 6000 platform (LC Bio Technology Co. Ltd., Hangzhou, China) following standard protocols.

### 2.6 Transmission electron microscope (TEM)

The OMVs stock solution was gently applied onto a carbon-coated support film and allowed to stand for 3 min. After removing the excess sample with filter paper, phosphotungstic acid stain was immediately added to the film. Following a 3-min staining period, the excess stain was again removed with filter paper. The carbon-coated film was then placed on filter paper in a dish and baked for 15 min. Finally, the morphology of the vesicles was observed using a transmission electron microscope.

### 2.7 Nanoparticle tracking analysis (NTA)

The outer-membrane vesicle solution was diluted 1:10,000 and thoroughly mixed. An appropriate volume was drawn into a 1.0 mL syringe, ensuring that any air bubbles were expelled. The concentration and size distribution of the vesicles were then measured using a nanoparticle tracking analysis (NTA) detector.

### 2.8 Statistical analysis

In this study, statistical analysis was performed using GraphPad Prism 9.0 software. Data are presented as mean ± standard deviation (SD). An independent sample *t-*test was used for comparisons between two groups, while one-way analysis of variance (ANOVA) was applied for comparisons among multiple groups. A *P*-value of < 0.05 was considered statistically significant.

## 3 Results

### 3.1 AKK supernatant enhances the secretion of MUC2 from goblet cells

**FIGURE 1:**
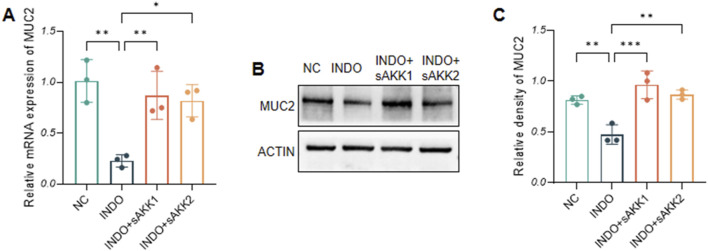
Effect of AKK supernatant on relative mRNA expression levels and protein level of MUC2. **(A)** The relative mRNA expression levels of MUC2. **(B)** The protein level of MUC2 measured by western blotting. **(C)** Quantitative analysis of the protein level of MUC2. Data are displayed as means ± SEM; *, **and *** indicate *P* < 0.05, *P* < 0.01 and *P* < 0.001.

**FIGURE 2:**
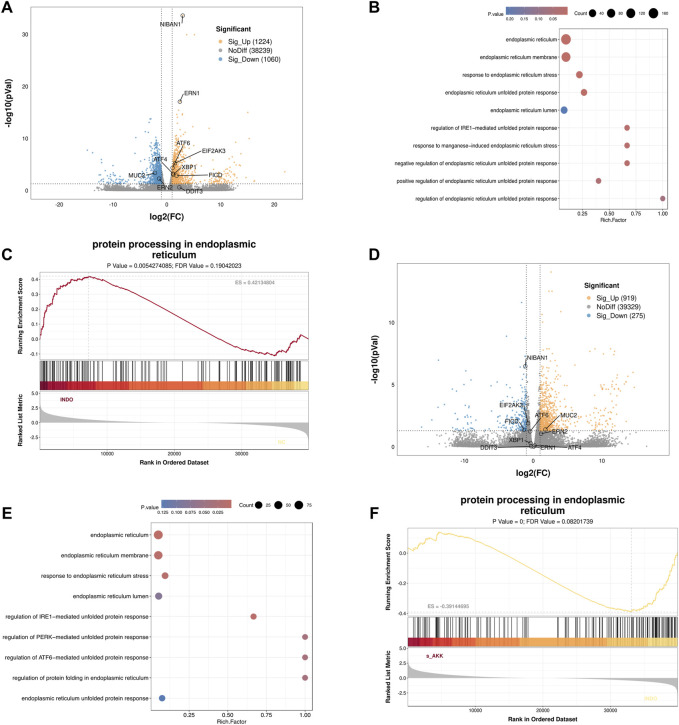
Transcriptome sequencing analysis. **(A)** Volcano enrichment plot for the differential genes of NC group, INDO group, n = 3. **(B)** GO enrichment scatters plot of NC group, INDO group, n = 3. **(C)** GSEA for protein processing in endoplasmic reticulum of NC group, INDO group, n = 3, |NES| > 1, NOM. p-val < 0.05, FDR. q-val < 0.25 is significant. **(D)** Volcano enrichment plot for the differential genes of INDO group, INDO + sAKK group, n = 3. **(E)** GO enrichment scatters plot of INDO group, INDO + sAKK group. **(F)** GSEA for protein processing in endoplasmic reticulum of INDO group, INDO + sAKK group, n = 3, |NES| > 1, NOM. p-val < 0.05, FDR. q-val < 0.25 is significant.

In an indomethacin-induced LS174T cell injury model, different concentrations of diluted AKK supernatant were used for treatment. qRT-PCR and western blot results showed that both the protein and mRNA levels of MUC2 were down-regulated in the INDO group compared to the NC group. However, compared to the INDO group, the expression of MUC2 was up-regulated in the INDO + sAKK1 and INDO + sAKK2 groups (Figures 1A–C). To further explore how AKK supernatant treatment directly regulates MUC2 secretion from goblet cells, transcriptome sequencing was conducted on cell samples from the NC group, INDO group, and treatment with INDO and 1:50 AKK supernatant (INDO + sAKK group). Using threshold criteria of fold change (FC) ≥ 2 or ≤ 0.5 and *q* value < 0.05 (|log2FC| ≥ 1 and *q* < 0.05), pairwise comparison between the INDO and NC groups identified 2,284 differentially expressed genes, including 1,224 up-regulated genes such as ER stress-related genes IRE1α (ERN1), XBP1, CHOP (DDIT3), and GRP78 (HSPA5), along with 1,060 down-regulated genes (Figure 2A). Gene Ontology (GO) analysis revealed that 21 of these differential genes were involved in biological processes related to the ER stress response, while another 21 genes were involved in the ER unfolded protein response pathway. Cellular component analysis showed that 171 differentially expressed genes were related to endoplasmic reticulum function (Figure 2B). Additionally, Kyoto Encyclopedia of Genes and Genomes (KEGG) analysis indicated that these differential genes are involved in protein processing in the endoplasmic reticulum, within the genetic information processing module class (Figure 2C).

Compared to the INDO group, a total of 1,194 differentially expressed genes were identified in the INDO + sAKK group, including 919 up-regulated and 275 down-regulated genes. The down-regulated genes included ER stress-related genes such as IRE1α (ERN1), XBP1, CHOP (DDIT3), and GRP78 (HSPA5) (Figure 2D). GO analysis revealed that two differential genes were involved in the IRE1-mediated unfolded protein response within biological processes, nine genes were involved in the ER stress response, 91 genes were associated with the ER cellular component, and 63 genes were linked to ER functions (Figure 2E). KEGG analysis showed that five differential genes were associated with protein processing in the endoplasmic reticulum within genetic information processing modules (Figure 2F). The transcriptomic results indicated that the expression of ER stress-related genes, including XBP1, IRE1α, CHOP, and GRP78, was up-regulated in the INDO group but down-regulated following AKK supernatant treatment. This suggests that AKK supernatant may have a regulatory effect on goblet cells by modulating endoplasmic reticulum stress.

**FIGURE 3:**
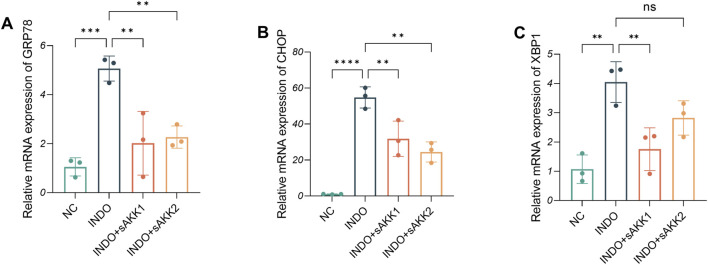
Effect of AKK supernatant treatment on ER stress. **(A–C)** The relative mRNA expression levels of CHOP, GRP78, XBP1, n = 3, one-way ANOVA. Data are displayed as means ± SEM; *, **and *** indicate *P* < 0.05, *P* < 0.01 and *P* < 0.001.

**FIGURE 4:**
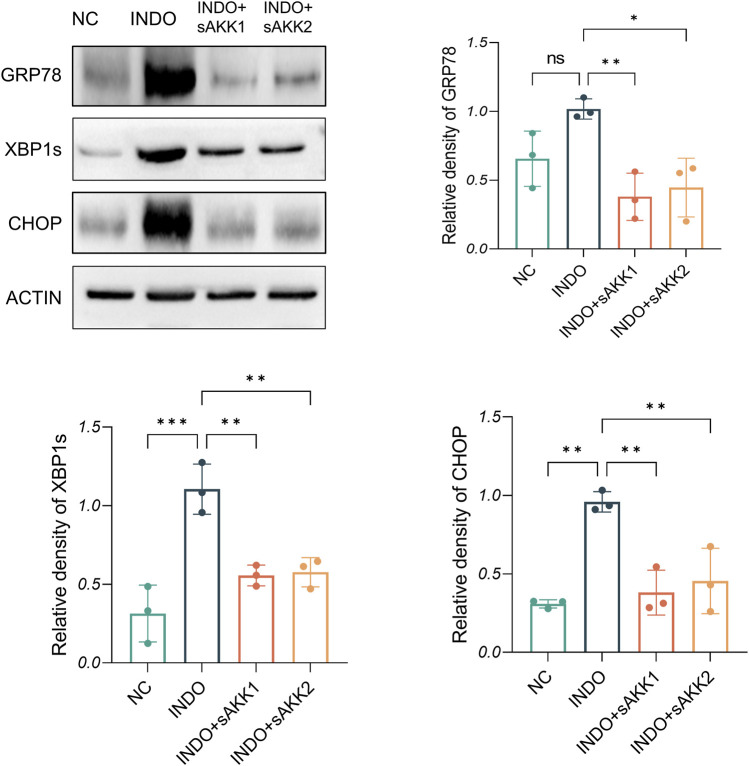
Effect of AKK supernatant treatment on ER stress. The protein level and quantitative analysis of CHOP, GRP78, XBP1s, measured by western blotting, n = 3, one-way ANOVA. Data are displayed as means ± SEM; *, **and *** indicate *P* < 0.05, *P* < 0.01 and *P* < 0.001.

Therefore, qRT-PCR and western blot analyses were performed to assess endoplasmic reticulum stress-related markers. The results showed an up-regulation of GRP78, CHOP, and XBP1 mRNA levels in the INDO group compared to the NC group, indicating indomethacin-induced ER stress. However, INDO + sAKK groups showed a down-regulation of GRP78, CHOP, and XBP1 mRNA levels compared to the INDO group, with no concentration-dependent effects observed between supernatant concentration and the degree of down-regulation (Figures 3A–C). Western blot results exhibited a similar pattern, with protein levels of GRP78, CHOP, and XBP1s following the same trend as their mRNA levels. The INDO group displayed an up-regulation compared to the NC group, while the INDO + sAKK1 and INDO + sAKK2 groups showed a down-regulation compared to the INDO group (Figure 4). These qRT-PCR and western blot analyses confirmed that AKK supernatant treatment may inhibit indomethacin-induced ER stress, thereby enhancing MUC2 secretion by goblet cells.

### 3.2 AKK-OMVs are the primary active ingredient found in the supernatant

**FIGURE 5:**
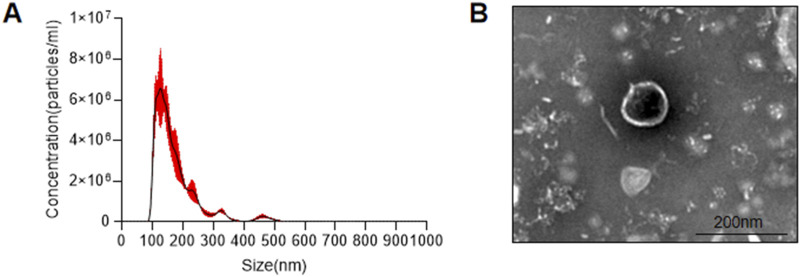
Size and structure of AKK-OMVs. **(A)** Size distribution of AKK-OMVs measured by NTA. **(B)** Morphological characterization of AKK-OMVs by TEM (scale 200 nm).

AKK supernatant is a substance with complex components. To further analyze which components in the AKK supernatant are responsible for its effects, two fractions were extracted: AKK-OMVs and the extracellular vesicle-depleted supernatant. NTA analysis was performed on the extracted substances, revealing that the average particle size of AKK-OMVs was 176.3 ± 1.5 nm (Figure 5A). TEM was used to examine the size and morphology of AKK-OMVs, showing that they possessed a uniform, double-layered vesicle-like structure (Figure 5B).

**FIGURE 6:**
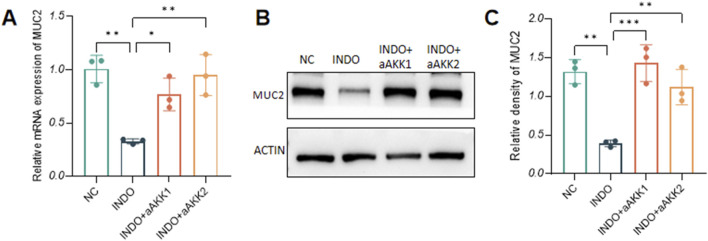
Effect of AKK-OMVs on gene and protein expression level of MUC2. **(A)** The relative mRNA expression levels of MUC2. **(B)** The protein level of MUC2 measured by western blotting. **(C)** Quantitative analysis of the protein level of MUC2. Data are displayed as means ± SEM; *, **and *** indicate *P* < 0.05, *P* < 0.01 and *P* < 0.001. **(A)**.

**FIGURE 7:**
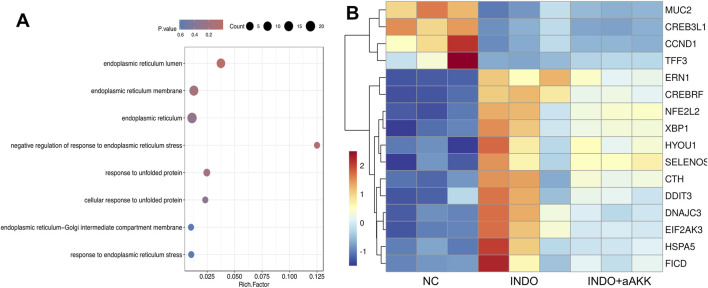
Analyst of AKK-OMVs on ER stress. **(A)** GO enrichment scatters plot of INDO group, INDO + aAKK group. **(B)** Heatmap of the differential expressed genes of INDO or INDO + aAKK compared to NC group, n = 3, Z-score.

**FIGURE 8:**
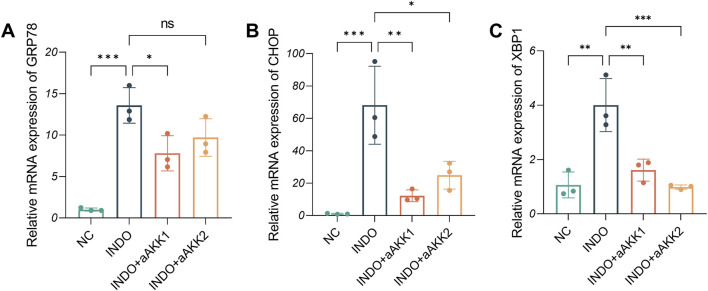
Effect of AKK-OMVs treatment on ER stress. **(A–C)** The relative mRNA expression levels of CHOP, GRP78, XBP1, n = 3, one-way ANOVA. Data are displayed as means ± SEM; *, **and *** indicate *P* < 0.05, *P* < 0.01 and *P* < 0.001.

**FIGURE 9:**
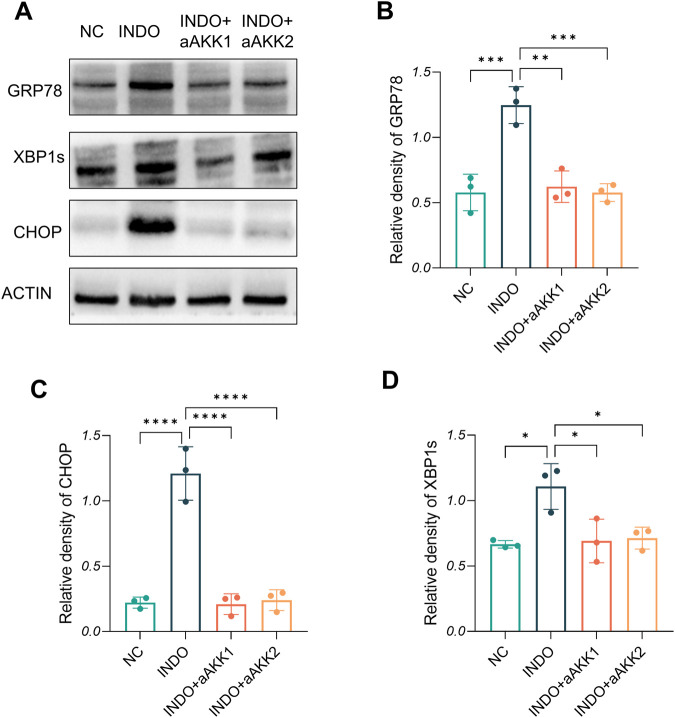
Effect of AKK-OMVs treatment on ER stress. **(A)** The protein level of CRP78, XBP1s , CHOP measured by western blotting. **(B–D)** Quantitative analysis of the protein level of GRP78, CHOP, XBP1s, n = 3, one-way ANOVA. Data are displayed as means ± SEM; *, **and *** indicate *P* < 0.05, *P* < 0.01 and *P* < 0.001.

When LS174T cells were stimulated with indomethacin, treatment with different concentrations of AKK-OMVs (INDO + aAKK1, INDO + aAKK2) were applied. qRT-PCR and western blot results demonstrated that, compared to the NC group, the mRNA and protein levels of MUC2 were decreased in the INDO group. However, this down-regulation was alleviated in both the INDO + aAKK1 and INDO + aAKK2 groups, where MUC2 gene and protein levels were up-regulated (Figures 6A–C). Transcriptome sequencing analysis was performed on cells from the NC, INDO, and INDO + aAKK groups. The results were consistent with those of the AKK supernatant treatment group, showing significantly up- regulation of genes in pathways related to the endoplasmic reticulum, ER stress response, and ER-associated degradation in the GO analysis (Figure 7A). The transcriptomic expression levels of ER stress-related markers GRP78, CHOP, and XBP1 showed a downward trend in the INDO + aAKK group compared to the INDO group (Figure 7B). qRT-PCR and western blot analyses confirmed that the expression levels of these ER stress-related markers were down-regulated in the INDO + aAKK group compared to the INDO group, at both the mRNA and protein levels (Figures 8A–C, 9A–D).

**FIGURE 10:**
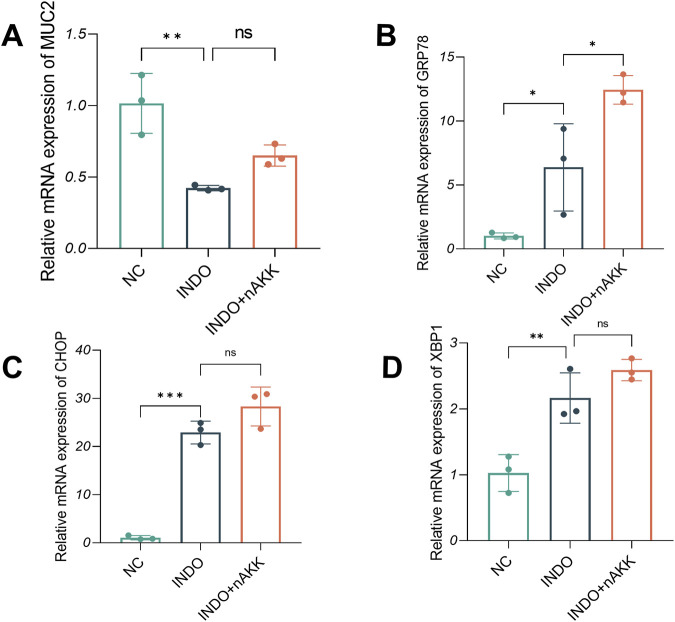
Effect of remove extracellular vesicles supernatant on MUC2, GRP78, CHOP and XBP1. **(A–D)** The relative mRNA expression levels of MUC2, CHOP, GRP78, XBP1, n = 3, one-way ANOVA. Data are displayed as means ± SEM; *, **and *** indicate *P* < 0.05, *P* < 0.01 and *P* < 0.001.

When LS174T cells were stimulated with indomethacin and treated with extracellular vesicle-depleted supernatant (INDO + nAKK), qRT-PCR results demonstrated a decrease in MUC2 expression in the INDO group compared to the NC group. However, no significant up-regulation of MUC2 expression was observed in the INDO + nAKK group (Figure 10A). This suggests that the extracellular vesicle-depleted supernatant did not alleviate the indomethacin-induced down-regulation of MUC2 expression. Furthermore, while the gene expression levels of endoplasmic reticulum stress-related markers GRP78, CHOP, and XBP1s were up-regulated in the INDO group, they did not show a down-regulation trend in the INDO + nAKK group (Figures 10B–D). Thus, it can be concluded that the extracellular vesicle-depleted supernatant does not inhibit indomethacin-induced endoplasmic reticulum stress.

Therefore, we propose that the extracellular vesicles present in the AKK supernatant may serve as the key active components exerting regulatory effects on goblet cells.

## 4 Discussion

This study demonstrated that AKK supernatant effectively mitigated indomethacin-induced reduction in mucin secretion by goblet cells through the inhibition of ER stress, with AKK-OMVs identified as a key active component in the supernatant. The findings revealed an increase in both MUC2 gene and protein expression levels in cells treated with AKK supernatant. Mechanistic investigations further indicated that AKK supernatant and AKK-OMVs reduced the expression of ER stress-related markers, such as CHOP, XBP1, and GRP78, in goblet cells, thereby inhibiting indomethacin-induced activation of ER stress pathways. These results suggest that AKK enhances MUC2 expression by modulating the ER stress pathway, while highlighting the regulatory role of AKK-OMVs in goblet cells.

Goblet cells, the primary secretory cells in the intestinal mucosa responsible for maintaining the mucus barrier function, comprise about 17% of the epithelial cells in the colonic epithelium and approximately 5% of the epithelial cells in the small intestinal epithelium (Nyström et al., 2021). These cells secrete large amounts of mucins (mainly MUC2), which are the main components of the intestinal mucus barrier. Due to their unique high secretion capacity, goblet cells are more susceptible to errors or abnormal folding of large mucin molecules compared to other epithelial cells (Naama et al., 2023). When misfolded or abnormally folded proteins accumulate to a certain threshold, ER stress occurs, activating the unfolded protein response (UPR) pathway to eliminate the misfolded proteins and maintain cellular homeostasis (Naama et al., 2023). Studies have shown that, compared to wild-type mice, mice with defects in MUC2 assembly and production have reduced mucin storage in their goblet cells. Furthermore, incomplete MUC2 assembly accumulates in the ER of goblet cells, triggering ER stress. Other studies have found that taurine deoxycholic acid (TUDCA), a bile acid derivative, can inhibit ER stress in wild-type mice, resulting in increased mucus secretion by goblet cells (Kihara et al., 2003). This suggests that ER stress may hinder mucin secretion in goblet cells.

Studies have shown that NSAIDs acts directly on the cell membrane, altering their biophysical properties (Lichtenberger et al., 2012), which leads to damage of intestinal epithelial cells and disruption of the intestinal mucus barrier, ultimately causing inflammatory damage to the intestines (Nyström et al., 2021). In this study, we found that NSAIDs can directly damage LS174T cells and reduce MUC2 secretion. In response to this injury, we used AKK supernatant as treatment and observed that it alleviated the indomethacin-induced reduction of MUC2 secretion from goblet cells. Transcriptomic analysis of cell samples revealed that ER stress-related genes (such as CHOP, GRP78, XBP1, and IRE1α) and biological processes (such as protein processing in the endoplasmic reticulum) were significantly upregulated in the indomethacin group. Further analysis at both the transcriptional and translational levels showed that the expression of ER stress-related markers (CHOP, GRP78, XBP1) was elevated in the indomethacin group, while these markers were down-regulated in the groups treated with different concentrations of AKK supernatant. However, no concentration-dependent inhibition of ER stress was observed. Therefore, these studies establish that MUC2 secretion in goblet cells is linked to the occurrence of ER stress, and that AKK supernatant may modulate ER stress-related pathways after indomethacin treatment in LS174T cells.

The bacterial supernatant refers to the metabolites produced by bacterial metabolic activities, as well as the soluble substances released when the bacteria break down after death. The soluble substances include bacterial outer membrane proteins, polypeptide chains, enzymes, and peptidoglycan derivatives (Tsilingiri and Rescigno, 2013; Postler and Ghosh, 2017). An increasing number of studies have found that probiotic supernatants contain various beneficial components that can play roles in anti-inflammatory, immune regulation, antioxidant, and other processes. AKK has garnered significant attention for its health-promoting effects. For example, AKK promotes intestinal barrier function by enhancing mucus secretion, increasing the number of goblet cells, and inducing stem cell differentiation into secretory cells (Cani et al., 2022). As research progresses, there is increasing focus on how AKK bacteria exert their effects and which components are responsible. In addition to the protein components extracted from the outer membrane of probiotics, extracellular vesicles derived from supernatant have gained attention. Many probiotic-derived extracellular vesicles have been shown to promote health in multiple ways. For instance, OMVs from *Escherichia coli Nissle 1917* can up-regulate tight junction proteins, enhance intestinal barrier function, and alleviate intestinal inflammation (Alvarez et al., 2016). Similarly, extracellular vesicles derived from *Lactobacillus rhamnosus GG* up-regulate Nrf2 expression, improve barrier function, and prevent alcoholic hepatitis (Gu et al., 2021).

Current studies have shown that AKK-OMVs can regulate mucosal immunity by inducing IgA responses, increasing colon goblet cell numbers, promoting mucin secretion, up-regulating tight junction protein expression, reducing colon epithelial damage, and modulating cytokine levels by increasing anti-inflammatory and decreasing pro-inflammatory cytokines to maintain intestinal homeostasis and relieve colitis (Toyofuku et al., 2023). In our study, we separated AKK supernatant into two components: AKK-derived outer membrane vesicles and non-outer membrane vesicle supernatant. These were used to intervene in cellular processes. The results showed that AKK-OMVs increased MUC2 expression and decreased ER stress-related signaling pathway proteins, effectively inhibiting ER stress. However, when the outer membrane vesicles were removed, these effects were not observed. This suggests that AKK-derived outer membrane vesicles, as an active component of the supernatant, can directly act on cells, inhibiting indomethacin-induced ER stress and alleviating the reduction in MUC2 secretion by goblet cells. However, our study did not delve deeply into the mechanism by which AKK-derived outer membrane vesicles regulate ER stress pathways. In future research, we plan to explore the underlying mechanisms in more detail and investigate the structure, composition, and characteristics of AKK-derived outer membrane vesicles.

In summary, we found that AKK-OMVs can directly stimulate goblet cells to promote MUC2 secretion. Our study provides a potential for further *in vivo* studies to confirm their protective effects against indomethacin-induced intestinal injury.

## Data Availability

The data presented in the study are deposited in the NCBI BioProject repository, accession number PRJNA1177854. The data can be accessed via the following link: https://www.ncbi.nlm.nih.gov/bioproject/?term=PRJNA1177854.
